# Applying ecological systems theory to juvenile legal system interventions outcomes research: a measurement framework

**DOI:** 10.3389/fpsyg.2023.1177568

**Published:** 2023-06-23

**Authors:** Kaitlin M. Sheerin, Regina Brodell, Stanley J. Huey, Kathleen A. Kemp

**Affiliations:** ^1^Department of Psychiatry and Human Behavior, Warren Alpert Medical School, Providence, RI, United States; ^2^Bradley-Hasbro Children’s Research Center, Rhode Island Hospital, Providence, RI, United States; ^3^Department of Psychology, University of Southern California, Los Angeles, CA, United States

**Keywords:** juvenile justice, intervention outcomes, children and adolescence, delinquency, ecological systems theory

## Abstract

Intervention research and development for youth in the juvenile legal system (JLS) has often focused on recidivism as the primary outcome of interest. Although recidivism is an important outcome, it is ultimately a downstream marker of success and is affected by changes in other domains of youths’ lives (e.g., family and peer relations, neighborhood safety, local and state-level policies). Thus, the present manuscript proposes the application of ecological systems theory to selecting outcomes to assess intervention effects in JLS intervention research to better capture proximal and distal influences on youth behavior. To that end, we first provide an overview of the strengths and limitations of using recidivism as an outcome measure. Next, the current application of social ecology theory to existing research on both risk and protective factors of JLS involvement is discussed, as well as existing work on assessing social-ecological domains within intervention studies. Then, a measurement framework is introduced for selecting pertinent domains of youths’ social ecologies to assess as intervention outcomes, moderators, and mediators. To facilitate this, we provide examples of concrete constructs and measures that researchers may select. We conclude with potential new avenues of research to which our proposed framework could lead, as well as potential limitations of implementing our framework.

## 1. Introduction

Each year, at least 700,000 youth enter the juvenile legal system (JLS) in the United States (Office of Juvenile Justice and Delinquency Prevention, 2021). Youth within the JLS represent a population vulnerable to marginalization, given that Black, Latinx, and gender and sexual minority youth are disproportionately arrested and incarcerated (Hirschtritt et al., 2018; Jonnson et al., 2019; Puzzanchera and Hockenberry, 2019; Puzzanchera and Hockenberry, 2021). Contact with the system has negative sequalae for these youth, who have documented difficulties with their behavioral health (Tolou-Shams et al., 2019; Kemp et al., 2020), academic achievement (Brown et al., 2008), peer relations (Miller-Johnson et al., 1999; Holloway et al., 2022), and family functioning (Tapia et al., 2018; Folk et al., 2020). As a result, the development and evaluation of efficacious interventions for youth in the JLS has remained a priority for researchers and policymakers alike.

Recidivism, defined as the commission of an offense after a youth has previously committed an offense (Blumstein and Larson, 1971), has most frequently been assessed through the use of official court records in intervention outcome studies (Olsson et al., 2021). There are several reasons that JLS intervention researchers have focused on recidivism. First, one of the main foci of the JLS is to enhance public safety and prevent further system contact (Office of Juvenile Justice and Delinquency Prevention, 2023). As such, understanding how intervention efforts impact recidivism is a system-level priority. Second, official recidivism data can be obtained with relative ease (e.g., through partnerships with legal systems) as compared to more in-depth in-person data collection (Harris et al., 2011). Third, assessing recidivism improves the ability to compare program effectiveness across states and systems, which, in theory, should also lead to the adoption of interventions beyond the jurisdiction they were originally tested in Sentencing Project (2010).

Although there are several strengths in using recidivism as the primary outcome of intervention effects, there are also several notable limitations. For example, official court records may underestimate the number of new crimes a youth may commit, with some estimates suggesting a thirty-to-one ratio between reported and actual crimes (Elliott, 1995). The assessment of recidivism has also varied across studies, with some researchers measuring self-reported delinquency, rearrest, reincarceration, or adjudication (Harris et al., 2011; Olsson et al., 2021). These disparate indices become even more varied when comparing across systems internationally (Fazel and Wolf, 2015). Caudill and Trulson (2022) also indicated the use of these varied assessments of recidivism among JLS-involved youth can lead to variable effect sizes across studies. In addition, recent work by Padgaonkar et al. (2021) indicates official rearrest rates may be influenced by racial bias such that Black JLS-involved youth are more likely to be rearrested than their White peers, despite committing fewer self-reported offenses prior to being rearrested. Thus, there are clear limitations to relying on recidivism as the primary outcome of intervention efficacy.

In JLS models aimed towards reducing recidivism (e.g., the Risk-Needs-Responsivity Model; Brogan et al., 2015), interventions are viewed as indirectly affecting recidivism through ameliorating a variety of “criminogenic” risk factors. In fact, recidivism is typically a distal outcome of most interventions and can be considered a downstream marker impacted by more proximal social determinants of health. The stated goal of many interventions for JLS-involved youth typically focus on antisocial and delinquent behaviors more broadly, often through changing individual youth factors (e.g., impulsivity, substance use) along with family, peer, school, and systems factors (Brogan et al., 2015). These changes are then thought to impact recidivism. In other words, recidivism is often the last domino to fall after several other areas have been addressed. To that end, it would appear more appropriate for researchers to use other indices beyond recidivism to better understand the more proximal and developmentally oriented effects interventions may have. Despite this, work by Schwalbe et al. (2012) indicate that measures beyond official recidivism records are inconsistently collected across studies. Further, juvenile risk assessments intended to assess multiple domains that together predict recidivism risk were not designed to be used in JLS intervention outcomes research. Juvenile risk assessments also rely heavily on user judgment and do not include youth and collateral perspectives (e.g., family members). As such, a shift towards more standardized and comprehensive assessment of these multiple domains in a youth’s life as part of understanding intervention outcomes is needed.

To promote the shift towards more proximal measures of intervention effects in JLS-focused research, the present article proposes using a classical developmental framework (i.e., ecological systems theory) to inform the selection of variables beyond recidivism. First, we provide an overview of Bronfenbrenner’s (1979) ecological systems theory and its application to the JLS. We next discuss how to use this model to select which variables might be considered intervention mediators, moderators, and outcomes. Importantly, we also include practical examples of pertinent constructs and measures regarding individual youth characteristics and elements of a youth’s social ecology. To conclude, we discuss areas for future work and possible challenges to implementation.

### 1.1. Ecological systems theory and the juvenile legal system

Bronfenbrenner’s (1979) ecological systems theory posits that youth behavior and well-being is influenced by the social systems in which youth find themselves embedded. These social systems interact with one another as well as the youth through interconnected subsystems. At the microsystem level, youth are directly impacted by their immediate social environment, such as family, peers, and teachers. The mesosystem is then comprised of interactions between these various subsystems (e.g., contact between parents and teachers), as well as with subsystems at other levels. The next level, the exosystem, is composed of individuals and contexts that are one step removed (e.g., neighbors, extended family, government organizations, the JLS), including those (e.g., caregivers’ workplace) with which youths’ family members interact At the macrosystem level, broader constructs, such as widely held cultural beliefs and laws are included. The chronosystem is the level most distal to the youth and consists of the broader sociohistorical context as well as changes over time. Bronfenbrenner also stressed that there was a complex interplay between all levels, which also reciprocally interacts with the youth. Moreover, ecological systems theory also focuses on the role that facets of a youth’s social ecology can play as both risk factors for and protective factors against the development of psychosocial concerns.

Much of the research on juvenile delinquency has been grounded in ecological systems theory, with several longitudinal cohort studies focusing on the link between youth risk and protective factors across several ecological levels and subsequent delinquent behavior and arrests. For example, in the *Pittsburgh Youth Study*, factors across the individual (e.g., impulsivity, ADHD symptoms, dealing drugs), family (e.g., poor parental supervision, use of physical punishment, lower levels of positive parenting), peer (e.g., exposure to deviant peers), and other more distal-levels (e.g., socioeconomic deprivation, living in a disadvantaged neighborhood) predicted delinquency (see Loeber et al., 1998 for a review). Similarly, findings from the *Pathways to Desistance Study* indicated that, among youth with serious arrest histories, neighborhood social organization was indirectly linked to delinquent behaviors through parenting practices and association with deviant peers (Chung and Steinberg, 2006). These studies represent just a handful of longitudinal, ecologically focused studies on JLS-involved youth and underscore the importance of assessing for multiple social ecological factors in understanding their relationship with outcomes for JLS-involved youth.

Despite the ecological systems focus of the literature on the development and persistence of delinquent behavior, intervention studies focused on JLS-involved youth inconsistently report on outcomes beyond recidivism (Schwalbe et al., 2012; Olsson et al., 2021). However, several studies of family- and community-based treatments for JLS-involved youth have reported microsystem-level outcomes, including parenting behaviors (Eddy and Chamberlain, 2000; Letourneau et al., 2009; Humayun et al., 2017), caregiver mental health (Borduin et al., 1995), global family functioning (Borduin et al., 1995, 2009), involvement with deviant peers (Eddy and Chamberlain, 2000), prosocial peer relations (Borduin et al., 2009), academic performance (Borduin et al., 2009), and school attendance (Leve and Chamberlain, 2007). Across these studies, race, gender, and socioeconomic status are often evaluated as potential moderators of treatment effects; however, more in-depth, nuanced measures of distal social-ecological factors (e.g., neighborhood deprivation) are rarely assessed as moderators. It is evident that multi-level indices can be collected as part of JLS intervention outcome research, but the collection of such data is not currently standard practice.

Viewed together, there is ample longitudinal work suggesting that social-ecological variables are linked with entry into the JLS, as well as continued system involvement. On the other hand, intervention studies appear to often neglect these facets of youths’ social ecologies when assessing for intervention effects. Thus, intervention studies often fail to inform us as to which risk and protective factors are addressed within the intervention. To bridge this gap, we recommend that investigators consider the ecological systems theory to inform the selection of intervention outcome variables.

### 1.2. Ecological systems theory as a framework for outcome selection

To put ecological systems theory into practice, researchers will need to apply a social ecological lens in selecting mediators, moderators, and outcomes pertinent to their intervention. To that end, there are several steps we recommend researchers take to guide their selection process. As an initial step, researchers should identify at which social-ecological level(s) their intervention occurs. For example, youth motivational interviewing interventions occur at the individual-youth level, family therapies occur primarily at the microsystem-level, and interventions seeking to change the JLS (e.g., juvenile drug courts) target the exosystem. In addition, some interventions such as multisystemic therapy target multiple levels of a youth’s social ecology. After establishing the social-ecological level(s) of the intervention, researchers should choose potential mediators that occur at the intervention level (e.g., assessing cognitive distortions for an individual youth cognitive behavioral intervention) and that are consistent with the intervention’s theory of change. Next, researchers should assess for *proximal* effects of their intervention ranging from variables at the intervention level down to facets of individual youth functioning (e.g., mental health). Given the focus that Bronfenbrenner’s (1979) model places on the interaction between various subsystems, we also propose that researchers include indices of more *distal* intervention effects through assessment at the next most proximal level beyond the one(s) at which the intervention is targeted (e.g., assessing variables at the mesosystem-level in a study of a family-based treatment). Finally, to select moderators, researchers should also assess for variables occurring at the other remaining distal social-ecological levels to gather information about the social-ecological contexts in which intervention effects occur. Moderators may also be selected from levels more proximal to the individual to inform tailoring of interventions to individual youth and families (e.g., age, race, gender, experiences of trauma and adverse events).

It is not practical for researchers to measure every possible facet of a youth’s social ecology in an intervention outcome study, largely due to sample size considerations and participant measurement burden. Some of the most distal, moderating influences on intervention effects (e.g., state-level JLS policies) may be best understood through meta-analyzes rather than a single outcome study—especially in cases of single site studies. Therefore, when weighing which outcomes, moderators, and mediators to assess for in such an outcome study, researchers must be selective in identifying facets of a youth’s social ecology that could both be reasonably measured and affected by the intervention. Moreover, the selection of these indices should be explicitly guided by the broader developmental literature on the link between social-ecological domains and youth behavior. For example, it seems reasonable to suggest that interventions focused on reducing aggressive behaviors may also improve peer relations at the microsystem-level, given prior work linking aggression with peer rejection in adolescence (Beeson et al., 2020).

Multisystemic therapy (MST; Henggeler et al., 2009b), provides a concrete application of this framework to JLS intervention outcome research in the body of work on social-ecological outcomes, mediators, and moderators. MST is a family-and community-based intervention in which interventions take place primarily within multiple domains in the youth’s microsystem (i.e., family, peers, school) and may also involve interventions at broader social-ecological levels (e.g., through targeting caregiver-school communication). Much of the evaluation research on MST has focused on youth recidivism and psychosocial functioning, as well as outcomes at the microsystem level (e.g., improved family functioning, reduced engagement with deviant peers; see Henggeler, 2011 for a review), with a handful of studies assessing for mesosystem-level variables (e.g., sibling and caregiver criminal involvement; Wagner et al., 2014; Johnides et al., 2017). Regarding mediators, a study found by Henggeler et al. (2009a) found that MST demonstrated favorable effects on antisocial behavior through reductions in deviant peer associations and improvements in caregiver discipline. Other work has established that improvements in family functioning mediate the link between MST and effects on long-term caregiver criminal involvement (Johnides et al., 2017), indicating that microsystem-level changes can impact the mesosystem for JLS-involved youth. Another study assessed for the moderating effect of neighborhood disadvantage on MST treatment effects and found that improvements in parental monitoring were linked with decreased problem behaviors only for youth and families residing in better neighborhoods (Robinson et al., 2015). This work highlights how facets of the exosystem can attenuate the effect that intervention-related changes in the microsystem may have on youth functioning. In sum, MST outcome research demonstrates that assessment across multiple ecological domains allows for a richer, nuanced understanding of intervention effects.

### 1.3. Overview of social-ecological constructs

Applying ecological systems theory to measuring intervention outcomes within the JLS poses a challenge for researchers in terms of selecting measures and constructs pertinent to their study. Thus, we provide examples of ecological systems-related constructs relevant to healthy development among youth in the JLS. In addition, we provide sample measures of each construct in Tables 1–6, with a focus on measures with validity for either youth within the JLS or adolescents in general. Across various social-ecological levels, we recommend leveraging a multimethod, multi-reporter assessment battery, consisting of youth report, caregiver report, reports from other relevant sources (e.g., teachers, juvenile justice personnel), and administrative and collateral records. Below, we discuss several constructs across social-ecological levels that researchers could consider assessing in intervention outcome studies.

**Table 1:** Measures of individual level constructs.

Measure name	Construct	# of items	Source	Method	Available languages	Access to measure
*Mental health*						
Child PTSD Symptom Scale for DSM-5 (CPSS-5; Foa et al., 2018)	Posttraumatic stress disorder diagnosis and symptom severity	27	Y	Self-Report, Interview	English; Spanish; Other languages	Online (Free) CPSS
Child and Adolescent Trauma Screen (CATS; Sachser et al., 2017)	Posttraumatic stress disorder diagnosis and symptom severity	20	Y; C	Self-Report; Interview, Caregiver Report	English, Spanish, Other languages	Online (Free) CATS
Kessler Psychological Distress Scale (K-6; Kessler et al., 2003)	Psychological distress	6	Y	Self-Report	English, Spanish, Other languages	Online (Free) Kessler6
The Mental Health Inventory (MHI-5; Berwick et al., 1991)	General psychological distress and well-being	18 (5)	Y	Self-Report	English, Spanish	Online (Free) MHI5
Suicidal Ideation Questionnaire – Junior (SIQ-JR; Reynolds, 1987)	Frequency of suicide ideation	15	Y	Self-Report	English	Online (Free) SIQ-JR
Self-Injurious Thoughts and Behaviors – Short Form (SITBI-SF; Nock et al., 2007)	Presence, frequency, and characteristics of self-injurious thoughts and behaviors	169 (72)	Y	Interview, Self-Report	English	Online (Free) SITBI-SF
*Substance use*						
AIDS Risk Behavior Assessment(ARBA; Donenberg et al., 2001)	Sexual behavior, drug/alcohol use, and needle use	32	Y	Interview	English	Online (Free) ARBA
The CRAFT 2.1 (Knight et al., 1999)	Substance-related risks and problems	9	Y	Interview, Self-Report	English, Spanish, Other languages	Online (Free) CRAFT
Risk Assessment Battery (RAB; Metzger et al., 1993)	Drug use practices and sexual behaviors	45	Y	Self-Report	English	Online (Free) RAB
Screening to Brief Intervention (S2BI; Levy et al., 2014)	For severity of substance use	7	Y	Self-Report	English	Online (Free) S2BI
Brief Screener for Tobacco, Alcohol, and other Drugs (BSTAD; Kelly et al., 2014)	Risky substance use and frequency of use	6–36	Y	Self-Report; Health Professional	English	Online (Free) BSTAD
*Life satisfaction*						
Satisfaction with Life Scale (SWLS; Diener et al., 1985)	Satisfaction with one’s life	5	Y	Self-Report	English, Spanish, Other languages	Online (Free) SWLS
Multidimensional Students’ Life Satisfaction Scale (MSLSS; Huebner, 1994)	Youths’ satisfaction across life domains	40	Y	Self-Report	English, Spanish, and Other languages	Online (Free) MSLSS
Brief Multidimensional Students’ Life Satisfaction Scale (BMSLSS; Seligson et al., 2003)	Youths’ life satisfaction in specific domains	6	Y	Self-Report	English	Online (Free) BMSLSS
Students’ Life Satisfaction Scale (SLSS; Huebner, 1991)	Global life satisfaction	7	Y	Self-Report	English	Online (Free) SLSS
*Risk behavior*						
2023 Middle School Youth Risk Behavior Survey Questionnaire (YRBSQ; Center for Disease Control and Prevention, 2022b)	Health behaviors	15	Y	Self-Report	Spanish, English	Online (Free) YRBSQ
2023 High School Youth Risk Behavior Survey Questionnaire (YRBSQ; Center for Disease Control and Prevention, 2022a)	Health behaviors	24	Y	Self-Report	Spanish, English	Online (Free) YRBSQ
Risk Assessment Battery (RAB; Metzger et al., 1993)	Drug use practices and sexual behaviors	45	Y	Self-Report	English	Online (Free) RAB
*Physical health*						
Children Health Questionnaire (CHQ; Landgraf et al., 1996)	Youth health-related qualify of life	87 (45)	Y; C	Self-Report	English and Other languages	Online (Free) CHQ
Children’s Global Assessment Scale (CGAS; Shaffer et al., 1983).	General Functioning	1	C	Interview	English, Spanish, Swedish	Online (Free) CGAS
*Acculturation*						
Acculturation Scale (Marin et al., 1987).	Acculturation for Hispanics	12	Y	Self-Report	English & Spanish	Online (Free) Acculturation Scale.pdf
Vancouver Index of Acculturation (Ryder et al., 2000)	Migrants’ orientations toward cultural and mainstream traditions	20	Y	Self-Report	English	Online (Free) Vancouver Index of Acculturation
Multidimensional Inventory of Black Identity (MIBI; Sellers et al., 1997)	Dimensions of Black identity and ideology	56	Y	Self-Report	English	Online (Free) MIBI
Native American Acculturation Scale (NAAS; Garrett and Pichette, 2000).	Level of involvement with Native American culture	20	Y	Self-Report	English	Online (Free) NAAS
*Prosocial behaviors*						
Weinberger Adjustment Inventory (Weinberger and Schwartz, 1990)	General social–emotional adjustment	7	45	Self-Report	English	Online (Free) Weinberger Adjustment Inventory
Strength and Difficulties Questionnaire (Goodman, 2001)	Behavioral and emotional difficulties	38	Y; C; T	Self-Report	English, Spanish, and Other Languages	Online (Free) SDQ

Y = youth; C = caregiver; T = teacher. Number in parenthesis indicate # of items on short form.

**Table 2:** Measures of microsystem level constructs.

Measure name	Construct	# of items	Source	Method	Available languages	Access to measure
*Family functioning*						
McMaster Family Assessment Device (Epstein et al., 1983)	General Family Functioning	12	Y; C	Self-Report	English	Online (Free) McMaster Family Assessment
Parent-Adolescent General Communication Scale (PAC; Barnes and Olson, 1985)	Parent–child communication	25	Y; C	Self-Report	English	Online (Free) PAC
*Parenting*						
Alabama Parenting Questionnaire (APQ; Frick, 1991)	Parenting practices	42	C	Self-Report	English	Online (Free) Copies of any publications using the APQ should be sent pfrick@uno.edu APQ
*Parental monitoring*						
Quality of Parental Relationships Inventory (Conger et al., 1994)	Quality of parental-adolescent relationships	42	Y	Self-Report	English	Online (Free) Quality of Parental Relationships Inventory
The Parental Monitoring Inventory (PMI; Steinberg et al., 1992)	Parental supervision strategies	9	Y	Self-Report	English	Online (Free) PMI
Parental Monitoring (P-Monitor; Stattin and Kerr, 2000)	Parental surveillance and knowledge of youths’ behavior outside the home	24	Y	Self-Report	English	Online (Free) P-Monitor
*Parental mental health*						
The Symptom Checklist (SCL-90; Derogatis, 1975b)	Psychological problems	90	Y; C	Self-Report	English, Spanish, and Other Languages	Online (Free) SCL-90
Psychiatric Diagnosis	N/A	Y/N	C	N/A	N/A	N/A
Brief Symptom Inventory (BSI; Derogatis, 1975a)	Psychological symptoms and distress	53	Y; C	Self-Report	English	Online (Free) BSI
*Exposure to Household Dysfunction, Adverse Events, and Trauma*						
Divorce	N/A	N/A	Y; C	Self-Report	N/A	N/A
Remarriage	N/A	N/A	Y; C	Self-Report	N/A	N/A
Death of a parent	N/A	N/A	Y; C	Self-Report	N/A	N/A
Transition from mother to father households	N/A	N/A	Y; C	Self-Report	N/A	N/A
One-parent family to a two-parent family (with a stepparent)	N/A	N/A	Y; C	Self-Report	N/A	N/A
Departure and reappearance of a parent or guardian	N/A	N/A	Y; C	Self-Report	N/A	N/A
Kinship (living with relative)	N/A	N/A	Y; C	Self-Report	N/A	N/A
Number of foster placements	N/A	N/A	Y; C	Self-Report	N/A	N/A
Adverse Childhood Experiences (ACES; Felitti et al., 1998)	Youth exposure to household dysfunction and child abuse/neglect	10	Y; C	Self-Report	English	Online (Free)
*Peer relations*						
Peer Conflict Scale (PCS; Marsee et al., 2011).	Dimensions of aggression	40	Y; C; T	Self-Report	English	Online (Free) PCS
The Peer Relations and Pro-Social Behavior Questionnaire (PSBQ; Rigby and Slee, 1992)	Bullying and anti-social behavior	20	Y; C; T	Self-Report	English	Online (Free) PSBQ
*Friendship*						
The Friendship Qualities Scale (FQS; Bukowski et al., 1994)	Quality of adolescents’ relationships with close friends	23	Y	Self-Report	English	Online (Free) FQS
The Quality of Relationships Inventory (Pierce, 1991)	Quality of relationships with close friends	25	Y	Self-Report	English	Online (Free) The Quality of Relationships Inventory
*Mentorship*						
Mentorship (Kelley and Lee, 2018)	Characteristics of the mentee-mentor relationship	6	Y	Interview	English	Online (Free) Mentorship
Mentor-Youth Alliance Scale (Zand et al., 2009)	Perceptions of youths’ relationship with a mentor	10	Y	Self-Report	English	Online (Free) Mentor Youth Alliance Scale
Mentor-Youth Relationships (Jucovy, 2002)	Quality of mentor-youth relationship	20	Y	Self-Report	English	Online (Free) Mentor Youth Relationships
*Microaggressions*						
The Racial and Ethnic Microaggressions Scale Youth Version (REMS-YV; Nadal, 2011)	Youths’ experiences of racial and ethnic microaggressions		Y	Self-Report	English	Online (Free) REMS-YV
*Gang membership/involvement*						
Esbensen Gang Involvement Survey (EGI; Esbensen et al., 2001)	Level of gang involvement	6	Y	Self-Report	English	Online (Free) EGI
Eurogang Youth Survey (Weerman et al., 2009)	Gang membership	21	Y	Self-Report	English, Spanish, and Other Languages	Online (Free) Eurogang Youth Survey
*Academic achievement*						
Grades	N/A	N/A	Y; C; T	Self-Report; Transcripts	N/A	N/A
Grade Point Average (GPA)	N/A	N/A	Y; C; T	Self-Report; Transcripts	N/A	N/A
State test scores	N/A	N/A	Y; C; T	Self-Report; Records	N/A	N/A
High school Completion	N/A	N/A	Y; C	Self-Report	N/A	N/A
Enrollment in higher education credits	N/A	N/A	Y; C	Self-Report	N/A	N/A
Credits earned	N/A	N/A	Y; C; T	Self-Report; Records	N/A	N/A
*School engagement*						
School Engagement Scale (SES; Fredricks et al., 2005)	Cognitive, emotional, and behavioral aspects of school engagement	15	Y	Self-Report	English, Spanish	Online (Free) SES
School is the Path Measure (Nurra and Oyserman, 2018)	Extent to which students perceive school as a pathway to success	5	Y	Self-Report	English & French	Online (Free) School is the Path
Harter’s Self Perception Profile for Children (Harter, 1985)	General self-worth and academic competence	36	Y	Self-Report	English	Online (Free) Harter’s Self Perception Profile
School attendance	N/A	N/A	Y; C; T	Self-Report Administrative Report	N/A	N/A

Y = youth; C = caregiver; T = teacher. Number in parenthesis indicate # of items on short form.

**Table 3:** Measures of mesosystem level constructs.

Measure name	Construct	# of items	Source	Method	Available languages	Access to measure
*Caregiver social support*						
Multidimensional Perceived Support Scale (MPSS; Zimet et al., 1990).	Social support	12	C	Self-Report	English	Online (Free) MPSS
*Caregiver-teacher involvement*						
Parent-teacher involvement questionnaire (Corrigan, 2002).	Parent and teacher involvement	26	C; T	Self-Report	English	Online (Free) Parent-teacher Involvement Questionnaire
*Caregiver-legal system involvement*						
Contact with probation officer (Vidal and Woolard, 2016).	Frequency and length of interaction with probation officer		C	Self-Report	N/A	Online (Free) Contact with Probation Officer
Caregiver/probation officer relationship quality (Vidal and Woolard, 2016).	Quality of parent interaction with probation officer	11	C	Self-Report	N/A	Online (Free) Caregiver/Probation Officer Relationship Quality
*Parent court involvement*						
Number of arrests	N/A	N/A	N/A	Court Records	N/A	N/A
Child welfare petitions	N/A	N/A	N/A	Court Records	N/A	N/A

Y = youth; C = caregiver; T = teacher.

**Table 4:** Measures of exosystem level constructs.

Measure name	Construct	# of items	Source	Method	Available languages	Access to measure
*Police youth engagement*						
Police Encounter Variable (Jackson et al., 2019)	Whether youth have ever been stopped by police	1	Y	Interview	English	Online (Free) Police Encounter Variable
Contextual Features of Stops (Jackson et al., 2019)	Context of youths’ encounter with police	6	Y	Interview	English	Online (Free) Contextual Features of Stops
Officer Intrusiveness (Jackson et al., 2019)	Behavior of police officer during interaction	7	Y	Self-Report	English	Online (Free) Officer Intrusiveness
Measure of Procedural Justice (Lind et al., 1997)	Perceptions of police fairness and equality	19	Y	Self-Report	English	Online (Free) Measure of Procedural Justice
Police Attitudes Toward Youth (Rabois and Haaga, 2002; Center for Applied Research in Human Development, 2008)	Police officers opinions and attitudes toward youth	10	C	Self-Report	English	Online (Free) Police Attitudes Towards Youth
Youth Attitudes Toward Police (Webb and Marshall, 1995; Fine et al., 2003)	Youths’ attitudes toward police officers	14	Y	Self-Report	English	Online (Free) Youth Attitudes Toward Police
*Exposure to violence*						
Conflict Tactics Scale-Parent/Child (CTS-P/C; Straus et al., 1998)	Physical and psychological maltreatment	3	Y; C	Self-Report	English	Online (Free) CTS-P/C
Survey of Children’s Exposure to Community Violence (Richters and Saltzman, 1990)	Youth exposure to threats in their community	13	Y; C	Self-Report	English	Online (Free) Survey of Children’s Exposure to Community Violence
Exposure to Violence (ETV; Selner-O’Hagan et al., 1998)	Youths’ exposure to violence	18	Y	Self-Report	English	Online (Free) Exposure to Violence
Expanded ACEs Questionnaire (Cronholm et al., 2015)	Youth’s exposure to community violence, crime, and discrimination	15	Y	Self-Report	English	Online (Free) Expanded ACES
*Neighborhood conditions*						
Neighborhood Qualities Measure (NQM; Mujahid et al., 2007)	Neighborhood safety and social cohesion	15		Self-Report	English	Online (Free) NQM
Neighborhood Conditions Measure (Sampson and Raudenbush, 1999)	Physical characteristics of youths’ neighborhood	21	Y	Self-Report	English	Online (Free) Neighborhood Conditions Scale
Neighborhood Safety (Winstanley et al., 2008)	Neighborhood disorganization	8	Y	Self-Report	English	Online (Free) Neighborhood Safety
*Caregiver workplace*						
The Perceived Employment Barriers Scale (PEBS; Hong et al., 2014)	Number and type of employment related barriers	20	C	Self-Report	English	Online (Free) PEBS

Y = youth; C = caregiver; T = teacher.

**Table 5:** Measures of macrosystem level constructs.

Measure name	Construct	# of items	Source	Method	Available languages	Access to measure
*Social status*						
MacArthur Scale of Subjective Social Status Youth Version (Goodman et al., 2001)	Youths’ perception of their family and social status	2	Y	Self-Report	English	Online (Free) MacArthur Scale of Subjective Social Status
MacArthur Scale of Subjective Social Status Adult Version (Adler et al., 2000)	Adults perception of their social status compared to others	1	C	Self-Report	English	Online (Free) MacArthur Scale of Subjective Social Status Adult Version
*Discrimination distress*						
Adolescent Discrimination Distress Index (Fisher et al., 2000)	Youths’ experiences of racial and ethnic discrimination	15	Y	Self-Report	English	Online (Free) Adolescent Discrimination Distress
*Economic stress*						
Current Economic Stress Scale: CESS (Shek, 2005)	Perceptions of economic stress	4	Y	Self-Report	English	Online (Free) CESS
*Racial trauma*						
Racial Trauma Scale (RTS; Williams et al., 2022)	Trauma symptoms arising from racial maltreatment	30	Y	Self-Report	English	Online (Free) RTS
UConn Racial/Ethnic Stress and Trauma Survey (UnRESTS; Williams et al., 2018)	Impact of racism-related experiences	48	Y	Interview	English, Spanish	Online (Free) UnRESTS
*Heterosexism*						
The Daily Heterosexist Experiences Questionnaire (DHEQ; Balsam et al., 2013)	Aspects of minority stress	50	A	Self-Report	English	Online (Free) DHEQ
The LGBT People of Color Microaggressions Scale (Balsam et al., 2011)	Impact of microaggressions related to racism and heterosexism	18	A	Self-Report	English	Online (Free) The LGBT People of Color Microaggressions Scale

Y = youth; A = adults.

**Table 6:** Measures of chronosystem level constructs.

Measure name	Construct	# of items	Source	Method	Available languages	Access to measure
*Life transitions*						
Transitional Impact Scale (TIS; Svob et al., 2014)	Life transitions	12	Y	Self-Report	English	Online (Free) TIS
*Intergenerational trauma*						
Adverse Childhood Experiences (ACES; Felitti et al., 1998)	Caregiver and Family adverse experiences	10	C	Self-Report	English	Online (Free) ACES
*Puberty*						
Pubertal Development Status Scale (PDS; Petersen et al., 1988)	Adolescent physical development	4	Y	Self-Report	English	Online (Free) PDS
Tanner Ratings (Tanner, 1962; Morris and Udry, 1980)	Onset and progress of puberty	5	Y; C	Self-Report	English	Online (Free) Tanner Ratings
*School transitions*						
Quality of Transition Instrument (Garner and Moots, 2018)	Student well-being after transitioning to a new school	24	Y	Self-Report	English	Online (Free) Quality of Transition Instrument

Y = youth; C = caregivers; T = teacher; A = adults.

Although there are numerous individual-level constructs pertaining to youth development and well-being, we select examples that we believe are especially relevant to youth in the JLS (see Table 1). For example, rates of mental health and substance use disorders are high among JLS youth, and problems often persist into adulthood (Teplin et al., 2002, 2021). Thus, assessing for these concerns should be considered by JLS researchers. In selecting indices of mental health, researchers can choose between more general indices (e.g., *The Mental Health Inventory*; Berwick et al., 1991) or more disorder or concern-specific indices (e.g., the *Self Injurious Thoughts and Behaviors Questionnaire*; Nock et al., 2007). To assess for substance use, there are measures which pertain to frequency of use (e.g., the *Brief Screener for Tobacco, Alcohol, and Other Drugs*; Kelly et al., 2014) or consequences of use (e.g., the *CRAFFT*; Knight et al., 1999). The latter may be particularly pertinent given work by Holloway et al. (2022) indicating that drug use consequences, rather than frequency, are related to recidivism for youth with early JLS involvement. Table 1 includes additional individual-level constructs and measures.

Measures at the microsystem level should involve assessments of a youth’s interactions with various subsystems (e.g., family, peers, school), as well as characteristics of those subsystems themselves (see Table 2 for measures). Within the family subsystem, there are several variables that may be especially important to consider. Prior work has established that several domains of parenting are linked with entry into the JLS, with findings from one meta-analysis suggesting that parental monitoring, control, and hostility are most strongly linked with delinquent behaviors (Hoeve et al., 2009). As an example, Frick’s (1991) *Alabama Parenting Questionnaire* is validated with youth in the JLS and has several subscales relevant to supportive and aversive parenting (i.e., positive parenting, caregiver involvement, monitoring/supervision, inconsistent discipline, and corporal punishment).

Traumatic events and other adverse experiences can also occur within a youth’s family and household at the microsystem-level. For example, adverse childhood experiences (ACEs; i.e., experiences of abuse and neglect, household dysfunction) may be important to consider in light of work linking ACES with recidivism among youth in the JLS (Wolff and Baglivio, 2017; Craig et al., 2020) and behavioral health in general among youth (Ballard et al., 2015). Given that youth in the JLS may have already accumulated several ACEs prior to study involvement, it could be the case that ACEs may be more well-suited as a moderator of intervention effects. If that were the case, then more in-depth measures of trauma exposure would be warranted. For example, the *Child PTSD Symptom Scale for DSM-5* (Foa et al., 2018) assess for both individual-level youth symptoms as well as exposure to interpersonal traumatic experiences. ACEs could also be evaluated more thoroughly through honing in on existing measures of relevant constructs. For example, living with a caregiver with a mental health disorder is characterized as an adverse experience. Thus, caregiver psychiatric diagnoses or global mental health (e.g., the *Brief Symptom Inventory*; Derogatis, 1975a) may also be important to assess, especially in light of caregivers of JLS-involved youth endorsing high levels of parenting stress and mental health concerns (Brown et al., 2018).

Peer relations and teacher influences are also important domains to consider assessing within a youth’s microsystem. Given the link between peer affiliation and entry into the juvenile and adult legal systems (Gatti et al., 2009), intervention researchers may consider assessing for both deviant peer associations (the *Esbensen Gang Involvement Survey*; Esbensen et al., 2001) or engagement with prosocial peers (e.g., the *Peer Relations and Pro-Social Behavior Questionnaire;* Rigby and Slee, 1992). Finally, because JLS-involved youth face many academic challenges (Brown et al., 2008), school records (e.g., grade and attendance records), and youth self-report of school engagement (the *School Engagement Scale*; Fredricks et al., 2005) may allow for an enriched understanding of this subsystem.

The mesosystem consists of interactions between various subsystems in a youth’s life, including between the family subsystem and other subsystems (see Table 3). For example, a recent study of Latinx JLS-involved youth reported that higher levels of caregiver school contact was linked with greater externalizing concerns; whereas, higher levels of positive caregiver school engagement was negatively associated with externalizing behaviors (Hoskins et al., 2021). This work highlights the need to for investigators to assess for caregiver-school relations in intervention studies. Thus, assessing facets of this relationship with caregiver and teacher-report measures such as the *Parent-Teacher Involvement Questionnaire* (Corrigan, 2002) may be useful. Assessing caregivers’ contact with members of the JLS could also be relevant, given work indicating that the quality of this relationship may be linked with youths’ success in complying with the terms of their probation (Vidal and Woolard, 2016). Caregiver’s own criminal legal system involvement should also be considered, with caregivers’ own engagement in illegal behavior posing as a well-studied risk factor for youth system involvement (see Besemer et al., 2017 for a review).

Several subsystems at the exosystem level (e.g., the legal system, caregivers’ workplaces, neighborhoods, communities) may be pertinent to JLS interventions, especially those which occur at a broader social-ecological level (see Table 4). First, given that youth in the JLS are inherently making contact with the legal system, their interactions with the system could be assessed. For example, there are measures of police-youth relations, including youth attitudes towards police (Fine et al., 2003) and police attitudes towards youth (Rabois and Haaga, 2002). Pertaining to neighborhoods and communities, the Expanded ACEs framework also would suggest that evaluation of household-level traumatic and adverse experiences should be broadened to include adverse experiences at the community and neighborhood-levels (Cronholm et al., 2015). Evaluating these community and neighborhood adverse experiences seems particularly relevant to JLS-involved youth, given work suggesting that youth within the system are more likely than their peers to reside in disadvantaged neighborhoods with high rates of crime and violence exposure (Chauhan and Reppucci, 2009; Wolff et al., 2018). Thus, it would likely be beneficial for investigators to use in-depth indices of neighborhood qualities in their work (e.g., *Survey of Children’s Exposure to Community Violence*; Richters and Saltzman, 1990). Beyond neighborhood and community contexts, caregivers’ interactions with the employment system in the United States, as well as with workplaces (for those who are employed), are also thought by Bronfenbrenner (1979) to play an important role in shaping healthy youth development. As an example, over half of primary caregivers in a sample of youth making first contact with the system reported that they were not currently employed (Yonek et al., 2019). Such work indicates the importance of assessing employment barriers for caregivers of youth within the system.

Constructs at the macrosystem-level include cultural values, beliefs, and laws, as well as broader cultural influences (see Table 5 for measures). Given that racial, ethnic, and sexual and gender minoritized youth make disproportionate contact with the JLS (Hirschtritt et al., 2018; Jonnson et al., 2019; Puzzanchera and Hockenberry, 2019; Puzzanchera and Hockenberry, 2021), the influence that experiences of racism and heterosexism exert on intervention effects for JLS-involved youth seems important to understand. More recently, researchers have begun looking at the effects of broader policy, laws, and broader cultural attitudes and beliefs on the efficacy of youth mental health interventions, which can inform intervention research within the JLS. In two studies, Price et al. (2021, 2022) accessed publicly available state-level data on explicit racial attitudes and cultural sexism to assess whether psychotherapies are less effective for youth residing in communities with higher levels of anti-Black racism and sexism, respectively. Findings from both studies indicated that psychotherapy was less effective for girls living in areas with higher levels of cultural sexism and for Black youth living in areas with higher levels of racism. Such innovative work suggests that researchers can leverage state- and community-level data on such cultural forces to determine its impact on intervention outcomes; however, in JLS outcome studies, this would require conducting the study at multiple sites. Thus, youth reports of their own experiences of discrimination and identity-based stress may also be important to document. For example, work by Martin et al. (2011) indicated that youth self-reported perceived discrimination, measured *via* the *Schedule of Racist Events* (Landrine and Klonoff, 1996), was directly linked with subsequent delinquency. Although less is known about experiences of discrimination and minority stress among sexual and gender minority youth within the JLS, studies indicate that experiences of minority stress among youth in the community are linked with behaviors which increase the risk of JLS involvement (e.g., substance use; Goldbach et al., 2014). Measures of discriminatory and traumatizing experiences related to youths’ identities could provide further contextual information for intervention efficacy and complement existing approaches of using static, demographic variables as intervention moderators.

Although the chronosystem represents a challenge in measurement, given the broad, sweeping constructs nested at this level, there are still several indices that may be feasible to assess (see Table 6 for measures). For youth involved in the JLS, the legal context seems particularly important to assess. Although laws are placed within the macrosystem, generational shifts within the legal system may have an effect on those seeking to do long-term follow-ups of their work. In fact, the shifts in the focus of the JLS noted earlier in the paper may moderate effects of interventions over time, indicating the need for either new interventions or changes to old ones (for a review of the pendulum swings of the JLS, see Cavanagh et al., 2022). In sum, although constructs at this level may present practical issues in assessment, there may be an important place for evaluating such constructs within longitudinal intervention outcome studies.

### 1.4. Future directions and implementation challenges

Our measurement framework is intended to serve as a springboard for an improved intervention science for JLS-involved youth, with the hope of guiding several lines of future inquiry. One such potential avenue would involve evaluations of adverse effects of interventions for youth in the JLS. Prior work has suggested that bootcamps, often used to treat conduct problems in JLS-involved youth, actually worsen conduct problems (Lilienfeld, 2007). Further, Rubenson et al. (2021) established that having law enforcement officers facilitate gang-focused interventions may lead to adverse outcomes under some circumstances. However, there are few, if any, outcome studies which have reported on adverse effects on social-ecological domains outside of the primary intervention target. Identifying which interventions reduce recidivism but lead to deterioration in other outcome domains could help inform the selection of which interventions to use with particular youth.

In future work, researchers can also leverage longitudinal outcome studies to understand more complex pathways of intervention effects. Specifically, the present measurement framework would likely allow for the use of serial mediation models (Hayes, 2017) to be able to measure how changes in one social ecological domain may lead to subsequent changes in other domains, and then ultimately, changes in youth recidivism. To date, most mediation studies have focused on intervention effects from models using single independent, dependent, and mediator variables. However, Deković et al. (2012) found that MST led to increases in parental competence, which, in turn, led to improvements in positive parenting, resulting in a decrease in youth externalizing behaviors. Such work indicates that facets of the youth microsystem interact with one another to yield improvements in youth behavior. As stated earlier, recidivism is often the last domino to fall in a longer causal chain. By identifying how interventions affect those dominoes earlier in the chain, we can gain a better understanding of what constructs to target and when to target them.

In general, the proposed framework would offer a shift from the standard assessment protocol within the field and, as with the implementation of any new practice, would not be without its tradeoffs. Researchers would likely have to contend with more practical issues, such as missing data and increased cost of paying participants to complete measures. Thus, our proposal represents a unique opportunity for researchers to engage in collaborations with agencies and organizations across youth’s social ecologies to facilitate data collection. Schools, JLS agencies, community organizations, and national organizations all collect data at broader social-ecological levels that could be used in outcome studies. For example, researchers can work with Unite Us and other organizations that gather large-scale data on local social determinants of health (Butler, 2021). Collecting such data would reduce participant burden and also allow for researchers to include variables not previously assessed in intervention studies in their work. Through such collaborations, JLS researchers could seek to use shared measures and protocols across studies, similar to the PhenX Toolkit used by NIH investigators (Hendershot et al., 2015). Further, conducting multisite intervention studies with larger sample sizes would also allow for investigators to look at more nuanced intervention effects (e.g., the effect of neighborhood context on outcomes). In sum, novel collaboration efforts will be essential to the implementation of this framework.

There are also several challenges to implementing such a framework. First, although we have identified several relevant constructs across youths’ social ecologies, there are likely indices that are not sensitive to change throughout treatment. Many of these constructs may not have been previously used in intervention studies. In fact, measures used across several intervention studies that fail to yield effects could be the result of an inability to detect change as opposed to a true lack of effect. Second, the extant literature for JLS-involved youth contains far more measures of risk factors than protective factors, which runs counter to both ecological systems theory and risk models in the JLS. Thus, there is great need to develop and validate measures which are sensitive to change throughout treatment and focus on protective factors for JLS-involved youth.

The use of a standardized assessment protocol is essential to being able to evaluate outcomes across youth. Despite this, applying a broader social-ecological lens makes it apparent that a one-size-fits all approach to measurement may miss important details. It could be the case that two youth participating in an intervention study both improve on the same measure of academic performance. However, one youth may improve due to increased school engagement at their school of origin, whereas another youth may improve due to moving to a better-fitting school. Such granular driving forces of intervention effects may be difficult to parse, given that researchers cannot be reasonably expected to measure every possible social-ecological variable. Further, typical sample sizes in JLS intervention research would not allow for such analyzes. Ultimately, these nuances are missed by using a standard intervention protocol in intervention research.

## 2. Conclusion

Shifting towards more universal assessment and report of JLS intervention effects on facets of youths’ social ecologies could lead to a more nuanced intervention science within the field. Researchers and those working within the JLS would be able to gain a better understanding of which social-ecological factors are able to be addressed by certain interventions. Further, collecting information regarding youth social environments is in line with JLS’s current focus on implementing interventions that can dually reduce system involvement *and* improve youth well-being (Cavanagh et al., 2022). Assessing broader social-ecological factors could lead to advances in personalized interventions through providing information on the circumstances under which particular interventions work best (e.g., which jurisdictions are best suited to implement certain interventions based on local policy and resources). Taken together, ecological systems theory can help to improve intervention outcomes research within the JLS.

## Data availability statement

The original contributions presented in the study are included in the article/supplementary material, further inquiries can be directed to the corresponding author.

## Author contributions

KS and RB contributed to the conceptualization, drafting, and editing of this manuscript. SH and KK contributed to the conceptualization, review of drafts, and editing of this manuscript. All authors contributed to the article and approved the submitted version.

## Funding

Funding for this manuscript was supported by the National Institute of Mental Health (5T32MH078788; R01MH129770) and the American Psychological Association’s Minority Fellowship Program.

## Conflict of interest

The authors declare that the research was conducted in the absence of any commercial or financial relationships that could be construed as a potential conflict of interest.

The reviewer CK declared a past collaboration with the author KK to the handling editor.

## Publisher’s note

All claims expressed in this article are solely those of the authors and do not necessarily represent those of their affiliated organizations, or those of the publisher, the editors and the reviewers. Any product that may be evaluated in this article, or claim that may be made by its manufacturer, is not guaranteed or endorsed by the publisher.
